# ‘Trying to battle a very slow version of the system that exists outside’: Experiences of waiting for healthcare in English prisons

**DOI:** 10.1177/13634593231195785

**Published:** 2023-08-28

**Authors:** Sue Bellass, Krysia Canvin, Laura Sheard

**Affiliations:** Manchester Metropolitan University, UK; Leeds University, UK; Keele University, UK; University of York, UK

**Keywords:** prison healthcare, qualitative research, temporal agency, waiting time

## Abstract

Prison has been described as the ultimate form of time-punishment – a place where time is no longer a commodity for individuals to spend, but is ordered by a system which symbolises its power through the control of segments of people’s lives. As such, a prison sentence epitomises the experience of waiting. Yet anticipating release is not the only form of waiting within carceral life; waiting for healthcare in its various forms also shapes people’s temporal experience. Drawing on interviews with 21 people who have lived in prison, this article describes how experiences of waiting for healthcare are mediated by expectation or hope, perceptions of the relationship between behaviour and healthcare access, and the consequences of waiting for care. Constraints on the autonomy of people in prison mean that waiting for healthcare differs in important ways from waiting for healthcare in the community, and can be perceived as an additional form of punishment. The experience of waiting for prison healthcare can affect physical and psychological well-being, and can in itself be understood as a pain of imprisonment.

## Introduction

Waiting, while an unexceptional occurrence in everyday life, is not an equitable phenomenon. Rather, waiting is intimately entwined with power, status and resources and hence both symbolises and reproduces the disparity between the powerful and the powerless (Adam, 1990; Auyero, 2012; Gasparini, 1995; Schwartz, 1975). Further, the experience of waiting is not uniform, but varies according to cultural norms (Hage, 2018; Hoffman, 2009; Wahidin, 2006), and can be mitigated by temporal agency; that is, individual autonomy to manipulate time through accessing cognitive and material resources to imbue waiting with meaning and productivity (Carr et al., 2014; Flaherty, 2011; Flaherty et al., 2020).

Waiting for healthcare adds a layer of complexity to waiting experiences. While actual wait times for healthcare are an object of scrutiny – monitored as an indicator of performance by clinical managers and regularly reported in the media (Carr et al., 2014) – the subjective experience of waiting for healthcare has received less attention (Fogarty and Cronin, 2008). Yet waiting for healthcare, which can be complicated by uncertainty, pain and suffering, grief for lost capabilities and existential fears relating to infirmity and mortality, is qualitatively different from other types of waiting, and may impact upon the experience of time itself. Studies suggest that people who lack temporal agency are more likely to experience wait times for healthcare as extended, challenging periods compared to those able to exercise some control to make the time pass at its normal pace or even more quickly than usual (Carr et al., 2014; Mats Sjöling et al., 2005).

The ability to enact such agency rests upon access to resources and to the temporal rhythms of the space people awaiting healthcare occupy. Prison represents a unique temporospatial environment, characterised by Foucault (1979) as a surveilled and regulated space using time to discipline and produce docile bodies, enacting punishment by depriving people of demarcated segments of their free lives. Importantly, research has suggested that the subjective experience of time in prison is wholly distinct from how time is operationalised through prison sentencing (Armstrong, 2014; Armstrong and Weaver, 2013; Carr and Robinson, 2022; Medlicott, 1999). For people in prison living according to a highly regimented timetable, there may be an uncomfortable duality of time both passing – marked by the ageing body and the loss of shared chronologies with loved ones – and standing still, as the undifferentiated regime merges one day with another (Armstrong, 2018; Cope, 2003; Crewe et al., 2020; Moran, 2012; Wahidin, 2002, 2006). The subjective experience of time in prison is deserving of attention, since evidence suggests that how and where time is spent in prison is intimately linked with mental health. Stephenson et al. (2021) found, for instance, that time spent outside the prison cell, and time spent in purposeful activity, were associated with better mental health and lower suicide risk.

Although there is a substantial body of literature exploring the subjective experience of time in prison, a relatively neglected dimension to date is the experience of waiting for healthcare. How waiting time while ‘doing time’ may be understood by people living in prison is an important area of enquiry in at least three ways. First, the prison population has high levels of health needs, and historically low levels of engagement with community health services (De Viggiani, 2006; Kouyoumdjian et al., 2019). Prison healthcare, though often under-resourced (House of Commons, 2018; Leaman et al., 2016), is charged with providing an equivalent standard of care to that of the community (Charles and Draper, 2012), and presents an opportunity to address health inequalities through the structured provision of services to a vulnerable population. Developing understandings about waiting for prison healthcare may elucidate opportunities to improve engagement. Second, exploring use of temporal agency in the negotiation of wait times may identify ways of making waiting more tolerable, lessening the ‘pains of imprisonment’ which are characterised by deprivation (Sykes, 1958) and uncertainty (Crewe, 2011). Finally, understanding waiting in the relational context in which it occurs, that is, with healthcare staff and prison officers, may offer insights into how security and health systems could be better integrated to ameliorate waiting experiences.

The data that follow were collected from a study on the quality of healthcare in prison in England. A persistent and spontaneously occurring narrative in virtually all the accounts was the experience of waiting: for appointments, for medication or test results to arrive, for risk assessments to be updated and for a response to complaints. This study explores the various forms of waiting time, offering insights into the subjective experience of those seeking healthcare in the prison setting.

## Method

This study aimed to understand perceptions of the quality of prison healthcare from the perspectives of those who have lived in English prisons. It formed part of a mixed-methods study funded by the National Institute for Health Research (NIHR HS&DR Ref. 17/05/26). We aimed to conduct semi-structured qualitative interviews with 20–25 adults (⩾18 years) who had been in receipt of prison healthcare. Recognising the additional burden that escorting a researcher in the prison estate would create, we opted to collect data from people living in the community who had been released from prison in the last 3 years. We aimed to construct a diverse sample of people to include people of different ages and ethnicities, people with long-term physical health conditions, and people experiencing issues known to be prevalent in the prison population such as substance misuse and mental health issues (personality disorders, depression, anxiety and psychosis).

Ethical approval was granted by the University of Leeds School of Medicine Research Ethics Committee on 17 July 2019 (ref. no. 18-093) and from Her Majesty’s Prison and Probation Service (HMPPS) on 24 December 2019 (ref. no. 2019-383) which permitted us to interview participants who were on probation.

Our recruitment strategy was designed prior to the Covid-19 pandemic. In the early stages of fieldwork, we conducted face-to-face recruitment of people who had lived in prison by spending time at non-profit, charitable and statutory services in the community. This relational groundwork, which enabled rapport-building with both agency staff and prospective participants, resulted in the successful recruitment of two participants in November 2019. However, restrictions caused by the pandemic led to a significant change in our strategy and a temporary hiatus in the fieldwork: HMPPS suspended approvals for all research between March and July 2020. When fieldwork recommenced, all contact with agencies and participants transitioned to remote methods, that is, email and telephone communication, in line with Government guidance. Consequently, all but the first two interviews were conducted by telephone.

Recruiting study participants became challenging due to myriad pandemic-related pressures faced by both recruiting agencies and potential participants. More time was allotted to recruitment to ensure that women and people with long-term physical health conditions, whose need for continuing care may lead to a particular experience with prison healthcare, were represented in the study sample.

We developed a topic guide through engagement with literature and consultation with people with lived experience, prison clinicians and the wider project team. Topics included health status, the prison healthcare process, experience of gaining access to medication and healthcare appointments, perceptions of constraints on prison healthcare, and relationships with healthcare professionals. The guide was employed flexibly and was adapted throughout data collection to incorporate new areas of inquiry.

Interviews lasted between 18 and 73 minutes (average length 35 minutes). All participants consented to the interview being audio-recorded and interviews were transcribed verbatim. Participants’ names were replaced with pseudonyms. Data were analysed using NVivo 12, using an initial line-by-line coding technique, and when waiting time was identified as a recurring theme in the narratives, all transcripts were re-coded and reanalysed thematically to draw out nuances of the experience of waiting.

## Findings

Twenty-one people (17 men and 4 women; see Table 1) were recruited for the study and interviewed between November 2019 and March 2021. The smaller number of women in the study reflects the gender disparity in the prison population: only around 4% is female (Sturge, 2021). Participants were aged 27–60, with a range of health conditions including mental health issues and substance misuse, and long-term physical health issues including diabetes, lupus and HIV. One person chose not to disclose their ethnicity; of those remaining, three identified as British Asian, one as Black African, one as Black British, one as White/Black Caribbean, and 14 as White British or White English. Thirteen of the sample had had one prison sentence in the last 5 years, while the remainder had had more than one, with three having had at least three sentences.

**Table 1.** Sample characteristics.

Pseudonym	Gender	Age	Ethnicity	No. of sentences in last 5 years	Prison category/categories**
Richard	M	35	White English	1	B, C
Jake	M	30	White British	1	B, C
Simon	M	43	White British	2	B, C
Robert	M	45	White British	1*	B
Stewart	M	41	White British	‘About 4’	B, C
James	M	27	White British	‘Many’	B, C
Amir	M	49	British Asian	2	B
Max	M	29	White British	1*	B, C
Robert	M	43	White British	‘Many’	B, C
Yousuf	M	44	British Asian	1*	B, C
Benjamin	M	39	White British	‘3 or 4’	B, C, D
Ollie	M	29	White English	1	B
Aadan	M	36	Black African	1	B
Jamie	M	38	White British	1	B, C
George	M	45	White British	1	B, C, D
Philip	M	46	White British	1	B, C
Steven	M	60	Black English	1*	A–D
Alesha	F	40	White/ Black Caribbean	2	Closed
Ariana	F	‘30s’	Did not wish to disclose	1	Closed/semi-open
Farah	F	39	British Asian	1	Closed/semi-open
Charlotte	F	48	White British	1	Closed

* Participant reported serving numerous sentences prior to the 5 year period.

** Male prisons are categorised from Category A (highest security) through to Category D (lowest security open prisons). Female prisons are categorised as closed (higher security) or semi-open/ open (lower security) conditions.

Emerging evidence suggests that the Covid-19 pandemic has significantly impacted waiting times for appointments for non-covid related health conditions (Canvin and Sheard, 2021). Virtually all of the participants in this study had been released from prison before 2020; hence the data relate to experiences of waiting unaffected by the pandemic.

It should also be noted that the organisation of prison healthcare is different in open compared to closed security conditions. The remit of open prisons is to prepare people for release and permits more autonomy. In terms of accessing healthcare, for instance, people in open conditions may be able to attend the healthcare unit unescorted for appointments with primary healthcare professionals, or to attend a hospital outpatient appointment alone. The data that follow relate to the experience of healthcare in closed conditions.

## Types of waiting for healthcare in prison

The analysis revealed different types of waiting for healthcare in closed prisons, differentiated by the prison timetable and the level of certainty – that is, whether a form of care was expected or hoped for by people living in prison, and the likely timing of the service. Thus, at one end of the spectrum, some forms of care were expected to happen at a given time according to the prison regime, while at the other, people hoped for care but with no knowledge of when, or if, it might be delivered.

## Waiting in expectation

Study participants described types of healthcare they were confident they would receive in prison, although the scheduling of the care could be known (in the case of reception screening and methadone provision) or not known (primary and secondary healthcare appointments).

There are regulatory forms of prison healthcare integrated into the prison regime that people entering prison receive. On arrival at an establishment, people attend a reception screening, followed up to a week later by a more detailed second screening. Screenings are typically conducted by a primary care nurse, who uses a standardised template to assess health needs, medications and vaccination status, and offer sexual health and blood borne virus screening. Although some people arrive with a supply of their medication from the sending prison, others need to have their medication confirmed by a healthcare professional prior to dispensing, which may, if a person is admitted at unsocial hours, or had no proof of their medication, lead to a hiatus in medication continuity.

Part of the admissions process is a risk assessment to ascertain whether people may keep a supply of their medication ‘in possession’ for self-administration in their cells, with medications deemed tradable amongst the prison population very unlikely to be given in possession. In such cases, and in those where in possession is not suitable for other reasons, the person will have to be unlocked and queue once or twice a day at a hatch to receive medication where administration is observed by a healthcare professional.

With the certainty that this healthcare would be delivered according to the timetable of the regime, participants’ narratives centred on the experience of waiting in the queue for the ‘hatch’, a small room with an opening through which healthcare staff can dispense medication. This form of institutionalised waiting was a significant experience for participants. While one person perceived it as an opportunity to meet friends housed in other units, in general, the queue was perceived to be an unpleasant experience; crowded, and with people behaving unpredictably, as one participant stated:

It was so much hassle that in the end I just gave up taking the meds. It’s not like going and standing in a nice queue, do you know what I mean, waiting? It’s all people jumping in and fights going on and drug dealing going on around you, and you are getting involved in stuff you don’t want to be involved in. . . It comes with a lot of violence. . . somebody next to you will get something over their head. . . it’s just a horrible thing. (George, 45)

Participants reported having to wait in the queue for anything from between 20 and 40 minutes, which could encroach on time needed for other activities during the short period of time unlocked from their cell. Participants reported sometimes having to make decisions about how to spend this time, which meant that medication could be missed:

In certain jails you’ve got 15 minutes to have a shower and get your meds. If you’re on the fourth landing you’ve got to get down to the [ground floor], by the time you get down and you get back up you’ve missed your shower or you’ve missed your meds. (Steven, 60)

Even though they may have to choose between competing priorities, the strict timetabling of everyday life in prison meant that participants expected to be able to join the medication queue at set times during the day. The timing of other forms of healthcare, however, though expected to happen eventually, are not known. In prison, people are able to request appointments with healthcare professionals by submitting an application – commonly referred to as an ‘app’ – which may be on paper or an electronic message sent from a kiosk. People are typically informed of their appointment the day before via a paper slip under their cell door or by their name being written on a whiteboard. Although two participants reported a wait of only a few days to see a GP or nurse, it was more common for waits to be reported as weeks or even months, comparing unfavourably to access to care in the community:

In prison it is long. You are waiting for ages to get seen. It could be something serious and you are waiting for a lengthy time. At the end of it, you don’t get treated equally in prison like you would do out here. (Yousuf, 44)

While waiting for appointments, several participants reported that they attempted to obtain updates from healthcare staff at the medication hatch to gain information, yet this strategy did not prove fruitful. Amir, 49, summarised participants’ perspectives when he said ‘they were just there to give you your meds and nothing else. If you had any concerns, they just told you “make an appointment with the doctor, we’re not here to listen to that”’. Further, even when in the system and waiting for the expected appointment, the healthcare may not take place as anticipated. They may find out about the appointment afterwards: ‘you get a slip through your door, “Your appointment was yesterday”’ (Steven, 60), may not be unlocked to attend the appointment, may be returned to the cell because of a security incident, or may arrive and wait in the healthcare unit only to have their appointment cancelled:

Sometimes you are sat in healthcare from 9:00 or even 8:30 in the morning and you don’t even get seen. ‘We have had to rebook your appointment.’ That adds to the stress even more because you have just sat in a room with no TV, no nothing. I understand prisoners should be punished. . . but you are just sat in a room with 10 or 15 other people for four hours with absolutely nothing to do. It is a tedious process. (James, 27)

Cancelled primary care appointments could have very debilitating consequences. Max, 29, who was taking methadone, reported that on arrival he was told he had an appointment with the doctor. He was then informed that the doctor had gone home, leaving him with withdrawal symptoms for 2 days: ‘it’s unnecessary suffering, isn’t it? I couldn’t move, I couldn’t walk, it was just horrible’.

Waiting for elective secondary care appointments adds a further layer of complexity since the person is not informed about their appointment in advance due to the risk of planned escapes. Participants in this study would sometimes become aware after the date that a secondary care appointment had been cancelled, either due to lack of escorting officers, or because healthcare staff had inadvertently mentioned the appointment. Appointments would then have to be rebooked, causing additional delay. Further, people may be transferred to prisons in other regions with outstanding hospital appointments which lengthens the wait for care. When people successfully attended outpatient appointments, waiting in public spaces handcuffed to escorting officers was described as a ‘very humiliating’ (Charlotte, 48) or “dehumanising” (Richard, 35) experience.

## Waiting in hope

While participants frequently reported that healthcare appointments had to be rearranged, they expressed a level of certainty that the care would eventually take place, albeit at an unknown time. For other forms of healthcare, however, there was more ambiguity about whether the care would ever take place, leaving participants to wait in hope rather than expectation. While methadone administration was generally perceived to be efficient once a person’s need for it had been established, participants reported that medication for other health conditions may not be at the hatch, or may be there but not be dispensed to them. One person noted that even long-term use of medication may not prevent this happening:

if the doctor prescribed a certain medication daily, twice a day, who’s the nurse to say ‘sorry, you can’t have it today’? I’ve been three or four times without the medication I was supposed to take on a daily basis. (Steven, 60)

At other times being moved within the prison, or being new to prison, might hamper medication continuity with distressing consequences:

I left the detox wing and my medication didn’t follow. . . I would have to queue up and these queues . . . they are massive, so I used to dread them queues and I think six days on the trot they didn’t have my prescribed medication. To be let down, that can do a lot of harm to someone mentally, especially in a place like that. (Jake, 30)

Others reported not being unlocked to join the medication queue, which they attributed to the prison officers not being aware that they were due to be unlocked, or to understaffing. For two participants with long-term conditions (cardiovascular disease and diabetes respectively), this engendered feelings of anxiety ‘you’re stuck in your cell worrying yourself sick’ (Steven, 60) and frustration ‘the prison staff didn’t know me, but at the end of the day if I am on the medication list I should be getting my medication’ (Aadan, 36). Missing medication can have serious consequences for the management or progression of a condition. In the case of antiretrovirals for HIV, for instance, missed medication can induce medication resistance. An HIV+ participant reported that he informed healthcare staff 8 weeks in advance that he would need more medication, but that it was not procured ‘until the very last minute’. While not missing a dose, he reported that, due to inefficiencies within the prison healthcare system:

I went something like 19 hours without any medication at all. . . I came very close to missing a dose and had to fight tooth and nail on two occasions to get that medication in time. I had to repeatedly go back to the meds hatch to keep asking ‘Have they arrived yet? Have they arrived yet?’ (Philip, 46)

There are several types of healthcare where there was uncertainty about both whether and when the care would be delivered. The first of these, access to mental health services, was the most common example across the participant accounts. Alesha, 40, for instance, compared an earlier prison sentence where she was able to be in a mental health unit, with a later one where she had to remain on the wing:

I was suffering in silence. I felt so suicidal it was horrible. . . Maybe they were full [on the unit], I don’t know. I ended up on the wing and I was very isolated, I was struggling, very bad. One time they said they were going to do a phone call, they said the Tuesday, and they didn’t, and I had to actually wait two and a half weeks.

Other participants related similar accounts. Simon, 43, described access to mental healthcare as ‘really, really, really bad’; for Max, 29, ‘terrible, terrible’. Farah, 39, reported making over 30 applications for mental health care without any response, while James, 27, observed that the impact of a lack of response after finally surrendering and asking for help ‘can make your position 1000 times worse’. Participants generally blamed poor resourcing within the system, rather than individual staff; Stewart, 41, for instance, observed that there were only three mental health nurses for 1500 people. Some participants reported that their methadone would be increased if they reported poor mental health, and that methadone increases – in Benjamin’s (39) terms ‘a liquid cosh’ – were used to keep people calm. Philip, 46, recounted that he did not feel listened to, stating:

Philip: Everything that was coming out of my mouth was just noise to them. You don’t really get help from the mental health team unless you start saying the right things.

Interviewer: What are the right things?

Philip: ‘I’m suicidal and I’m going to harm myself’, or people will literally stand there with a blade in front of officers. I felt so desperate. I was referred to the mental health team and I had one appointment and the other two got cancelled. . .It’s people like me who end up dying in prison. It’s the quiet ones who suffer.

Gaining access to dentists, opticians and physiotherapists, who only run a limited number of clinics could also, according to most participants, lead to waits of 6–7 weeks or longer, or may never take place. Robert, 45, reported that he had submitted an application for the dentist at the start of a fifteen-month sentence, and received the appointment a week before release. Further, he noted ‘I’ve seen people with massive abscesses in their mouths, and they’re told ‘you have to put your name down to see the dentist’, and it could be up to 6 months’. Similar waits were reported for other visiting clinicians, with George, 45, noting ‘by the time your specialist appointment came round your injury was cleared up, it was that long, or you had got a disfigurement’.

Less common in the dataset were narratives of waiting to leave prison to get treatment for injuries at Accident & Emergency. Though few in number, however, these were vivid accounts. Max, 29, had his jaw broken, which he described as ‘separated at one side and hanging at the other side, my face was literally hanging off’, and recounted that he had been told to join a queue amongst people waiting for paracetamol. While waiting, he ‘worried what was going to happen to my face’, and noted that it took around an hour to get to hospital because of the prison’s security system.

Yousuf, 44, who had a broken hand, and James, 27, a broken leg, reported a wait of 2 or 3 days, which James interpreted as an additional form of punishment for his behaviour:

There was a bit of an altercation on the wing. I snapped my tibia in two places and it took them three days to get me out to the hospital. I wasn’t prepared to tell them [prison officers] what had happened. I was punished for not saying who it was. . . I believe that’s why the healthcare was withdrawn.

Two other forms of indeterminate waiting were waiting for test results and waiting for a response to complaints about healthcare. Three participants reported not being given test results for several weeks or even months. Richard, 35, awaiting the results of a mole biopsy for 6 months, was eventually given a letter dated a few months previously. George, 45, submitted an app to discover test results on a suspected hernia, but did not receive information until a hospital appointment 6 months later.

For three participants, the frustration caused by the uncertainty of whether healthcare would be delivered was exacerbated by raised expectations, as Farah, 39, explained:

Don’t offer something if you can’t provide that service because it’s almost as if they dangle carrots in front of you and then take them away. It’s horrible. It really is horrible.

## Waiting and behaviour

Several participants perceived that there was a relationship between behaviour and healthcare access: poor behaviour or frequent requests for help were perceived to irritate prison officers. Ollie, 29, who had worked in a prison as an operational support grade (a role supporting trained prison officers) prior to his conviction, observed that:

if you were a well-behaved prisoner and didn’t cause the officers any trouble, but you went with a bad back, you seemed to get a new mattress within a week and painkillers the next day. If you are a chronic self-harmer who takes his anger out on officers and is rude to healthcare staff, but threaten to kill yourself tomorrow, you get to see healthcare in two weeks’ time.

For Charlotte, 48, and James, 27, healthcare such as timely in-possession assessments was associated with privilege, earned through good behaviour on the wing. James recounted an experience where, when having toothache while in a position of privilege, he was able to access the dentist the same day, whereas when he was on ‘basic’ he had to wait for 2 weeks, leading him to conclude that ‘I do believe that they [prison officers] have a certain amount of influence on how quick you can and can’t be seen’. He reported that a good relationship with officers may result in them contacting healthcare directly rather than an app having to be submitted. Conversely, he noted:

I have had stitches left in my face for 12 days when they were supposed to be in there for seven because there was numerous events over the space of a week that kept happening. I am not sitting here and trying to say I was an angel in prison. I made life difficult for them, they made life difficult for me.

Participants frequently alluded to being treated as a collective rather than as individuals, with assumptions made that they were drug seeking rather than having legitimate clinical needs. Steven, 60, highlighted the importance of building a relationship with officers in terms of gaining access to healthcare. ‘In remand you don’t really get time to get a rapport with the screws [prison officers] and therefore . . .they think you’re a malingerer until they get to know you’. Sometimes participants found it difficult to explain why some people would get better access to treatment than others, irrespective of need, leading to suffering ‘she is screaming for help, but she’s just being ignored’ (Farah, 39) or even death ‘if they’d have opened the door quicker, they’d have saved them’ (Steven, 60). Charlotte, 48, noted:

there was somebody else on my wing who had a very painful problem with her gallbladder, but she was just left to deal with her pain. They make these kind of almost playing God decisions. . . what do you put that down to? I don’t know, I actually don’t know.

## Waiting and agency

The most common action people took to try and reduce their waiting times was persistent behaviour, even though they perceived that this risked further delays. Philip, 46, noted that ‘you have to be very, very persistent’. Sometimes it’s almost like you’re wasting their time. Max, 29, who was in ‘absolute agony’ with toothache, reported having to keep ‘forcing it and forcing it and forcing it and forcing it’ to get a dentist’s appointment.

This sense of powerlessness may be more broadly embedded in experiences of feeling ‘lower class’ (Simon, 43) or ‘second-class citizens’ (Max, 29). Capturing a sentiment expressed by several of the participants, Max continued ‘the punishment’s being there, isn’t it, not what they can do to you while you’re there’.

Several participants compared their agency in the prison setting to the community, suggesting that they had more control over access to healthcare in the community. Amir, 49, stated that ‘there might be a waiting time, but you know where you stand, you get to talk to the people you want to talk to’. Similarly, Aadan, 36, noted that there is a greater degree of interaction between patient and professional in the community; for Steven, 60, and James, 27, emergency appointments could easily be accessed in the community compared to in prison:

They tell you ‘it’s the same on the street, you have to wait, blah blah blah. Nonsense. Once you’re on the street you realise that’s nonsense. You phone up the dentist and you get it [an emergency appointment] within 24 hours. That very rarely happens in jail. (Steven, 60)

A barrier to the use of agency appeared to be lack of knowledge of what caused delays or cancellations. The accounts were strewn with instances of being given very limited information about why things happened. Jamie, 38, for instance said:

you put in a request, they would send a reply ‘it is being dealt with’ and then you just either would not hear from it again or it would take forever and that was the story I heard time and time and time and time. . .everybody on the wing told me the same story.

For Aadan, 36, and Steven, 60, pressing their buzzer to be unlocked for medication was not reported to be successful. Steven recalled being told ‘Stop ringing your bell. It’s an emergency bell.’ ‘Well it’s an emergency, I haven’t had my medication.’ ‘That isn’t an emergency.’

Several participants reported submitting complaints about healthcare in prison. Although two people reported receiving an apology for not being unlocked for medication or treatment, most recounted that did not receive any response, and doubted the effectiveness of the complaints process: ‘I’m sure they get chucked away, you never get a reply. . . we all joke about it’ (Jake, 30) and ‘the officers will just get it and bin it’ (Max, 29). For Richard, 35, the responses were not timely enough for him to appeal decisions:

it will be every excuse used to try to make it as awkward as possible for you to complain, even the response coming back, so that if you appealed it they would say you have appealed it out of time even though you didn’t get a response on time in the first instance.

Some participants undertook creative actions to try and manage their waiting times. Farah, 39, struggling with her mental health, submitted an app to see the GP saying she couldn’t eat due to stomach cramps; however, although referred by the GP for ‘about the eighth time’, she did not see a mental health professional. Philip, 46, who was refused a phone call to his parents to secure his HIV medication, asked a cellmate with access to a talk text phone due to deafness to make the call on his behalf. Other people, with less sense of agency, withdrew into their own psychological or physical space. Alesha, 40, facing an urgent mental health need, who said she ‘wasn’t one for speaking up to the officers’, self-isolated, not engaging in association time with others. Similarly Simon, 43, who reported he was struggling with ‘massive anxiety’ reported that he only left his cell to go for showers: ‘that’s the only way I could deal with it’.

The most drastic act of agency to reduce waiting times for healthcare mentioned by participants was self-harm or attempted suicides. Simon (43) noted ‘if you threaten [suicide], that’s not enough, you’d have to actually attempt to do it’, while Farah, 39 stated it was ‘the only way to get attention’. For James, 27, self-harm was the only action he could take to access support: ‘the only time I have had an instant response from the officers and the mental health team is when I have cut myself. It is too late by then. It could be too late by then’.

## Discussion

This study has illuminated the significance of waiting for healthcare in prison, offering insights into how people have experienced different forms of waiting for healthcare in the context of the prison regime. Waiting can be predicated on expectation, or reliant on hope, the gradual erosion of which may culminate in enacted despair. For people in prison, the dual identity of ‘waiting prisoner’ and ‘waiting patient’ could overlap: waiting for healthcare could be perceived as an additional – and unjust – form of punishment.

Waiting may therefore be conceived of as an additional pain of imprisonment, particularly when it is not known when or if the care will be delivered. Crewe’s (2011) exposition of the pains of uncertainty helpfully highlights the effects of unpredictability on people in prison. For Crewe, the inconsistent application of rules ‘expands the psychological territory that power occupies’ (p. 513), engendering a sense of insecurity. Similar insights can be gained from the broader literature on waiting. Withholding knowledge from the waiting person is, according to Bendixsen and Eriksen (2018), an expression of power: ‘the punitive aspect of waiting consists of ensuring that the person who waits is kept in the dark regarding both the length and the outcome of the wait’ (p. 98). Some of the accounts in this study indicate high levels of uncertainty around waiting for healthcare, creating anxieties about the potential effects of delayed care on physical injuries or long-term health conditions. Thus being made to wait, not being told how long the wait is likely to be, or why it exists, continually reproduces the asymmetry in the power and status of people in prison and the system itself.

Waiting for healthcare in prison thus combines physical and psychological dimensions of experience, in contrast to Sykes’ (1958) feted analysis of the pains of imprisonment which was founded upon a distinction between the physical punishment people in prison experienced in the past through ‘brutality’ and ‘neglect’ (p. 64), and the psychological punishment experienced in the present through deprivations of liberty, goods and services, heterosexual relationships, autonomy and security. In accounts in this study, the physical and the psychological are intimately entwined, with anxiety and frustration induced by waiting for healthcare with a physical injury or an ongoing health condition needing treatment. As Fuchs (2005) noted, the ill or injured body disrupts taken-for-granted understandings of time, bringing awareness to the interval between need and fulfilment. That the accounts of participants precipitated a focus on waiting time as a key concept within the research indicates that this awareness is keenly felt, and in this least autonomous of environments, the ability to attenuate the interval is constrained.

Much of the broader literature on the experience of waiting draws attention to the degree of agency and intentionality the waiting person has, and whether they wait passively, confident that the looked-for outcome will materialise, or engage in individual or collective actions to influence the wait (Bendixsen and Eriksen, 2018; Hage, 2009). In prison, however, time essentially becomes de-commodified and objectified. For Wahidin (2006), time is ‘no longer a resource to be *used, spent* or *saved* but an object to be managed’ (p. 106, original emphasis) while for Crewe et al. (2020), people in prison are ‘reconciling themselves to a state of protracted limbo’ (p. 317). Nonetheless, they argue, people seek to customise their experience of prison time despite the constraints of the carceral timetable. The findings of this research are consistent with both studies; there is evidence that people use their limited autonomy to try to improve their situation through individual actions. This study also reveals that people may continue with repeated actions allowed, if not always gratified, by the regime – making enquiries at the medication hatch, submitting complaints – even when experience has shown these strategies to be fruitless. This suggests either that hope for a different outcome prevails, or that frustration motivates people to continue to take system-tolerated actions. Additionally, there was some evidence of creative responses to waiting: leveraging the telecommunications privilege accorded to someone with impaired hearing, and fabricating a physical health need to gain access to the GP to discuss mental health issues. Future research might explore creativity and prison healthcare waiting times in more depth.

Time cannot be separated from space, and the uniqueness of the carceral environment directs researchers to consider how the two dimensions intertwine (Armstrong, 2018; Medlicott, 1999; Moran, 2012). This study found that the spatial dimension of waiting was more prevalent in participant narratives when the outcome of the wait was deemed more predictable, even if it did not actually transpire. People may wait in a slow-moving yet chaotic queue at the medication hatch, contained in their cell waiting for an appointment, in a holding cell in a healthcare unit, or at the prison gates to be taken to Accident and Emergency. For participants in this study, the cell was depicted as a place of frustration, anxiety, respite or despair; the medication queue could be unruly or tedious; the holding cell monotonous, and the wait to leave for Accident and Emergency services frustrating. Having to leave prison to wait in public spaces brings another – often ignominious (Abbott et al., 2020) – dimension to waiting for healthcare.

Everyday waiting is a banal experience that has received less attention than waiting as emblematic of structural disadvantage (e.g. Auyero, 2012; Jeffrey, 2010). Waiting for healthcare in the prison setting perhaps provides the ideal locus for gaining insights into the interplay between subjective experience and system-induced waiting, since the person is constrained by a regimented system whose purpose is to dislodge the symbols and privileges of adulthood: liberty, self-determination and temporal autonomy (Medlicott, 1999; Sykes, 1958). Further, in trying to access healthcare in a secure setting such as a prison, people are faced with navigating not one but two complex systems with differing, at times competing, priorities (Powell et al., 2010). Understanding how waiting for healthcare in prison is experienced within the broader landscape of waiting that characterises prison life gives important insights into a ‘pain of imprisonment’ that incorporates physical and psychological dimensions.

### Limitations and further research

Through conducting research on the quality of prison healthcare, this study has cast light on a hitherto under-researched subject; that of the experiences of waiting for healthcare in prison. As these data began to be collected during interviews, the topic guide was adapted to enquire specifically about the experience of waiting. Nevertheless, the importance of this experience, and the impact of waiting on participants’ physical and mental health, suggests that this phenomenon deserves to be the sole focus of research studies. Further, while we explored actions people took to try and attenuate the wait, we focussed less on actions that people employed to make wait times pass more quickly.

Given the relatively small number of women in the sample, conducting a thorough analysis to compare the experiences of women and men waiting for healthcare in prison was problematic. In light of important scholarship that draws attention to the gendered experience of authority in prison (Bosworth, 1999; Bucerius and Sandberg, 2022; Liebling, 2009), we acknowledge this limitation in our study, and contend that this as an important area for future research.

People waiting for healthcare in the community may benefit from informal support from family and friends (Drageset et al., 2011, Khatri et al., 2012). With only limited contact with informal networks, people in prison face constraints in gaining support from family and friends during what can be a challenging life event. Further research could explore experiences of waiting for healthcare within this broader relational context.

### Implications for policy and practice

Participants’ accounts are suffused with a lack of knowledge of the causes of delays and missed appointments. While providing information about waits for forthcoming external appointments is legitimised as a security risk, providing more information about internal appointments, cancellations or test results, may reduce levels of anxiety and frustration. Greater collaboration between healthcare and security systems may reduce actual wait times for internal and external care. Further, finding ways of imbuing healthcare wait times with meaning and purpose may offer a protective effect on mental health. Encouraging a greater sense of control, part of the focus of the health-promoting prisons agenda (Woodall, 2020) could incorporate such an objective.

Actual waiting time data is routinely collected from English prisons through the Health and Justice Indicators of Performance, a set of quality indicators for prison healthcare. Theoretically, then, accountability for wait times is built into the healthcare commissioning process. However, these data are not in the public domain, and not a subject for public discourse, in contrast to wait times for non-carceral healthcare which are regularly reported in the media (Carr et al., 2014). Greater transparency regarding prison healthcare wait times would enable care equivalence to be more closely scrutinised; an important step in addressing health inequalities experienced by prison populations.

## Conclusion

Hage (2018) encourages us to consider the question of whether waiting is a universal or culturally-specific phenomenon. We have argued here that the temporal norms of the institution of prison shape experiences of waiting for healthcare in particular ways. The regimentation of the entry screenings and methadone queue remove doubt that healthcare will be provided: here, fulfilment of the medical need is expected according to the timetable of the system. In light of this predictability the subjective gaze turns to enduring the institutional waiting represented by the queue. Where the outcome of waiting is uncertain, the interval between need and fulfilment engendered by the ill or injured body can create space for anxiety, frustration and ultimately despair to flourish.

Other studies on waiting for healthcare have found that indeterminate waits can create feelings of powerlessness and frustration (Carr et al., 2014; Mats Sjöling et al., 2005). When considering the effects of waiting for healthcare in prison, however, we must be cautious in making comparisons with people in the community, who have greater autonomy, status and resources, are more likely to be able to bring meaning and purpose to waiting, contact healthcare staff and obtain analgesic medications to relieve pain. Further, waiting for healthcare in prison is diffused within the more general ‘waiting out’ (Reed, 2011, p. 530) that defines prison life, and, for some, creates a sense of the use of infirm corporeality as an opportunity for an additional means of punishment.
